# Deep learning assisted XRF spectra classification

**DOI:** 10.1038/s41598-024-53988-z

**Published:** 2024-02-14

**Authors:** Velibor Andric, Goran Kvascev, Milos Cvetanovic, Sasa Stojanovic, Nebojsa Bacanin, Maja Gajic-Kvascev

**Affiliations:** 1 VINCA Institute of Nuclear Sciences, University of Belgrade, National Institute of the Republic of Serbia, Belgrade, 11000 Serbia; 2 University of Belgrade, School of Electrical Engineering, Belgrade, 11000 Serbia; 3 Department of Informatics and Computing, Singidunum University, Belgrade, 11000 Serbia

**Keywords:** Deep learning, Autoencoder neural network, XRF spectra, Dimension reduction, AI classification, Pigments, Canvas paintings, Electrical and electronic engineering, Characterization and analytical techniques

## Abstract

EDXRF spectrometry is a well-established and often-used analytical technique in examining materials from which cultural heritage objects are made. The analytical results are traditionally subjected to additional multivariate analysis for archaeometry studies to reduce the initial data’s dimensionality based on informative features. Nowadays, artificial intelligence (AI) techniques are used more for this purpose. Different soft computing techniques are used to improve speed and accuracy. Choosing the most suitable AI method can increase the sustainability of the analytical process and postprocessing activities. An autoencoder neural network has been designed and used as a dimension reduction tool of initial $40 \times 2048$ data collected in the raw EDXRF spectra, containing information about the selected points’ elemental composition on the canvas paintings’ surface. The autoencoder network design enables the best possible reconstruction of the original EDXRF spectrum and the most informative feature extraction, which has been used for dimension reduction. Such configuration allows for efficient classification algorithms and their performances. The autoencoder neural network approach is more sustainable, especially in processing time consumption and experts’ manual work.

## Introduction

Over the years, EDXRF spectrometry has become well-established and widely used to analyze materials from which cultural heritage objects were made^1^. The common procedures for quantifying the analytical results are used mostly for metallic^2,3^ and ceramic objects^4^. However, some analyses can only result in a qualitative interpretation highly dependent on a time-consuming expert involvement. The most complex analytical problem is the analysis of the materials of painted layers onto different supports, which remains in the domain of qualitative results interpretation, whether related to material identification^5^, mapping materials distribution^6^, or attribution based on the used materials^7^. The results obtained using the EDXRF analytical technique are multivariate whether used as selected informative peaks related to the elements in the material composition or as full spectrum. Quantitative or qualitative results are analyzed using multivariate^8^ or pattern recognition techniques^9^. Depending on the purpose, such an approach led to developing of more or less complex, robust, and sustained procedures^10^. The present study focuses on the problems of time attribution of the paintings, based on the classification models developed on trustworthy information about the authorship. Classification models based on the EDXRF spectral data were chosen since the technique is most used in everyday practice.

Firstly, it is necessary to perform dimension reduction of the analytical results. Different chemometric dimension reduction techniques showed good potential for the purpose^10^, and deep learning methods were now employed to examine its potential and advantages, especially in sustainable performances. The main characteristic of the chemometric approach is that developing models is time-consuming and needs high expert involvement. To reduce these specified factors, the neural networks (NN) were designed and trained in this study to reduce the input dataset dimensions more sustainably. The fast development and intensive applications of deep learning techniques and neural networks in different aspects of science and technology found their applications in spectrometry. Among the problems that were successfully addressed is dimension reduction. Particularly, nonlinear dimension reduction techniques gave significantly better results in various fields of application^11–15^. Autoencoders find applications in various domains, such as image and signal denoising, dimensionality reduction, anomaly detection, and feature learning. They are fundamental in learning efficient data representations by compressing the input information into a reduced-dimensional space and then reconstructing it, aiming to capture the essential features of the input data. Some studies showed that autoencoder neural networks enable speed and robustness by effectively reducing input data dimensions and obtaining advanced features^13,16^.

On the other hand, it was shown that in contrast to classical machine learning chemometric methods, deep neural networks can learn critical patterns from raw spectra, which involves fewer human factors in preprocessing and feature selection, ultimately enhancing model accuracy, and robustness^17^. Consequently, the prevalence of deep network architectures is rising, given that experts are no longer necessary for feature engineering, leading to sustainable spectra treatments^18^.

The application of convolutional neural networks (CNN) led to the conclusion that a large amount of data is required for CNN training, so there is a need to generate synthetic spectra and apply the transfer learning technique. The results are good on synthetic spectra but significantly more modest on real spectra. Thus, the sustainability of this approach is called into question^19,20^. The use of autoencoder neural networks has the potential to overcome the disadvantages mentioned above^9,21^, which was the starting point for the study presented here.

This study aims to explore the possibilities of the autoencoder neural network as the dimension reduction technique based on the reconstruction of the XRF spectra. The dimension reduction was performed to examine the structure of the input dataset, meaning to explore possible variance in the period of paintings creation and find low-dimensional space suitable for undoubted attribution. The number of layers and the number of neurons per layer were changed during neural network design. At the same time, different activation functions were employed to get the highest between-class separability in the reduced space. This paper presents the feasibility of ascertaining the optimal hyperparameters through extensive experiments tailored to address the abovementioned issue. The raw, natural logarithm and square root transformed spectral data were used to test the neural network design and efficiency to ensure reliable procedure. The class separability was measured using Bhattacharyya’s distance. This parameter has the highest value for squared-rooted transformed input spectral data with seventy neurons in the encoder/decoder layers and two in the bottleneck layer. Moreover, class coherence was the best-preserved during dimension reduction for the same neural network design.

## Results and discussion

### Pigment composition analysis

The EDXRF measurements allowed the detection of the chemical elements in the painted layer materials and the most precise pigment identification possible. The identified pigments have been listed in Table 1, together with the corresponding paintings.

**Table 1:** Pigments that were used throughout the period of painting creation.

Pigment	Period
Yellow ochre	All three periods
Chrome yellow	Early (KI-14) and middle (KI-8)
Naples yellow	Early (KI-13 and KI-14) and middle (KI-8)

**Figure 1:**
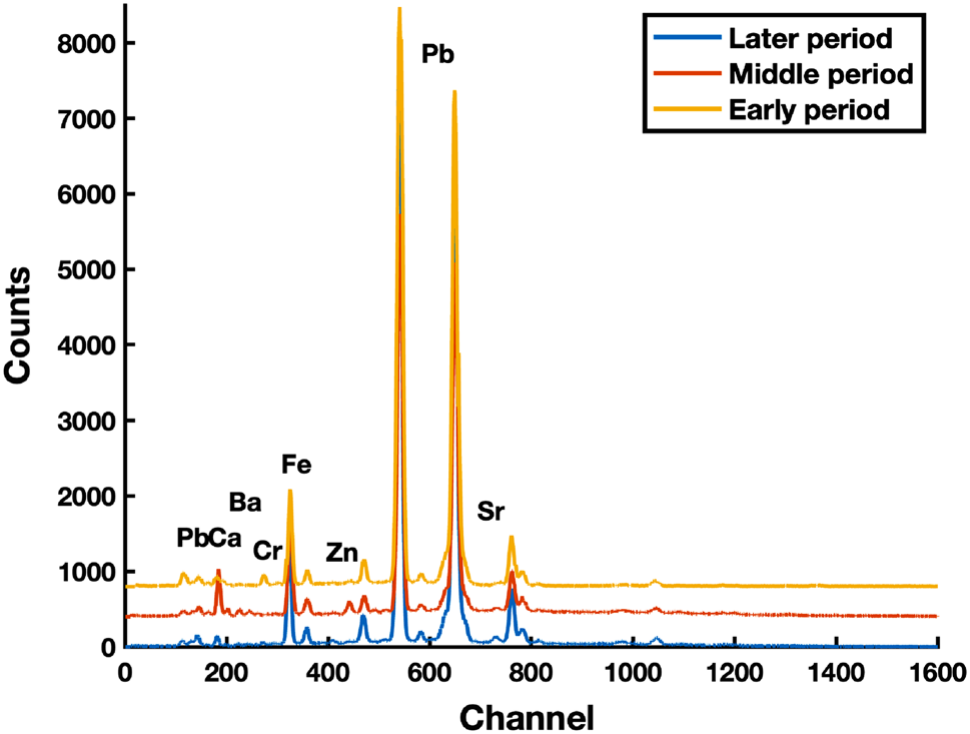
The reduced EDXRF spectra collected at yellow-colored spots on paintings from three different periods of creation.

Besides pigments listed in Table 1, lemon yellow was detected in several analyzed spots, while even the usage of massicot pigment can’t be excluded. At least five chemically different yellow pigments were used and can be found on most analyzed paintings, regardless of the creation period. This result means that the variance of the information regarding the time can be small in the input dataset. The EDXRF spectra selected from three periods of painting creation are presented in Fig. 1. As can be seen, the lead peaks are the most intensive, which can mask the relevant information about the specificity of the period. Those two findings led to the conclusion that deep learning methods can help reveal informative data for time attribution of the paintings regarding the chemical composition of the used pigments.

### The autoencoder neural network hyperparameters selection

**Figure 2:**
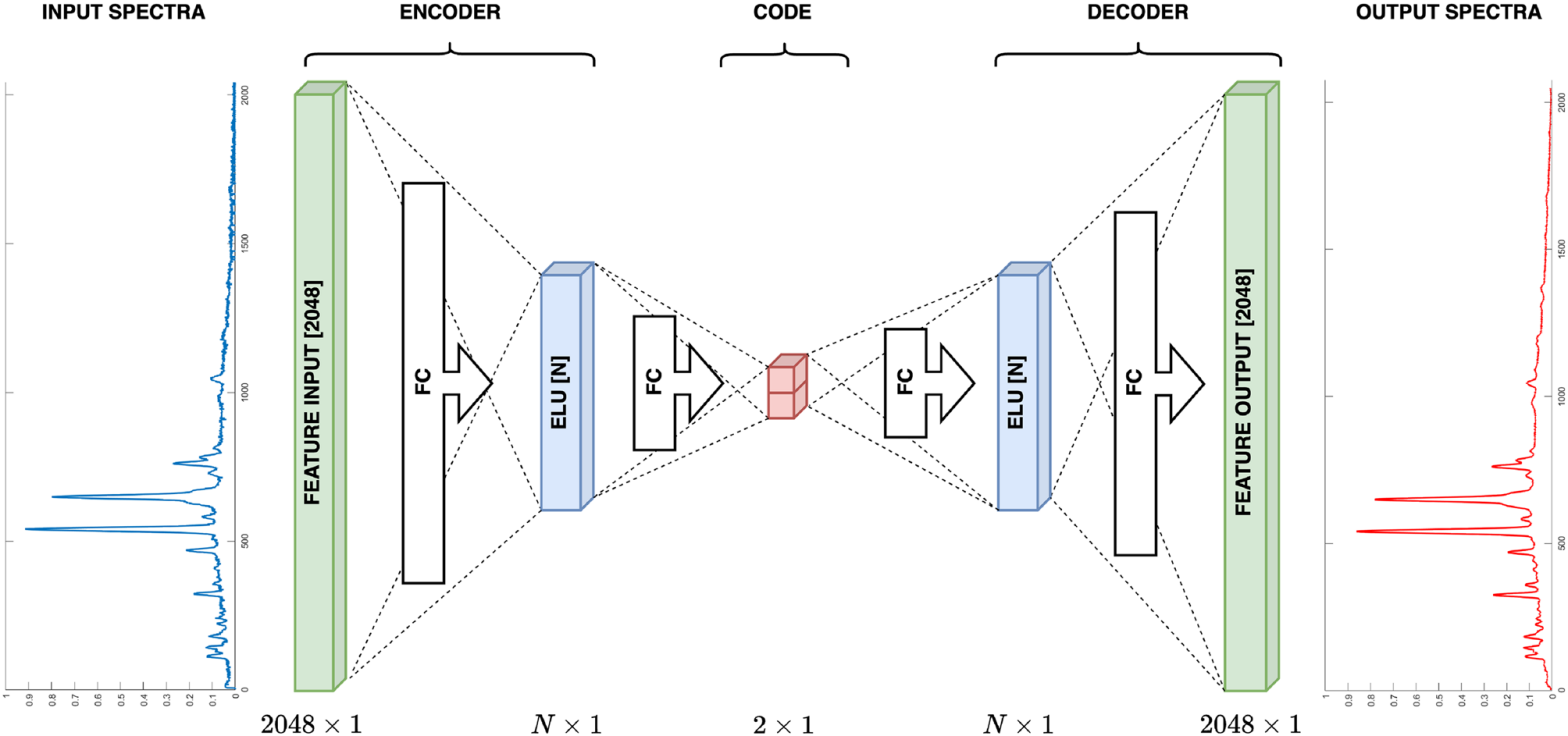
The stacked autoencoder neural network (SAENN) structure.

The choice of hyperparameters (number of layers and neurons in the encoder/decoder layers and activation functions) enables autoencoder neural network design to be an efficient dimension reduction procedure. Meeting the goal of autoencoder neural network efficient training, the bottleneck layer dimension was chosen to be two to ensure visual control of the approach. The outputs of this layer represent the $x_1$ and $x_2$ coordinates in a reduced space, so the visualization is unambiguous. Additional evaluation was conducted to determine the optimal number of layers between the input and bottleneck layers to enable the network to reconstruct the input spectrum effectively. The goal was to create a network that meets the criteria while minimizing its dimensions to mitigate overtraining/overfitting risks. Extensive simulations demonstrated that incorporating just one additional layer rendered the network sufficiently complex to achieve satisfactory reconstruction quality. While more intricate networks also displayed promising results, they were dismissed due to the potential for overtraining. Exponential Linear Unit (ELU) activation functions enhanced training speed and accuracy^22^, along with a variable training rate initially set at $LR=0.001$. The schematic representation of the stacked autoencoder neural network (SAENN) design (expanded vanilla structure autoencoder neural network) is shown in Fig. 2.

### The autoencoder neural network hyperparameters training and dimension reduction

**Figure 3:**
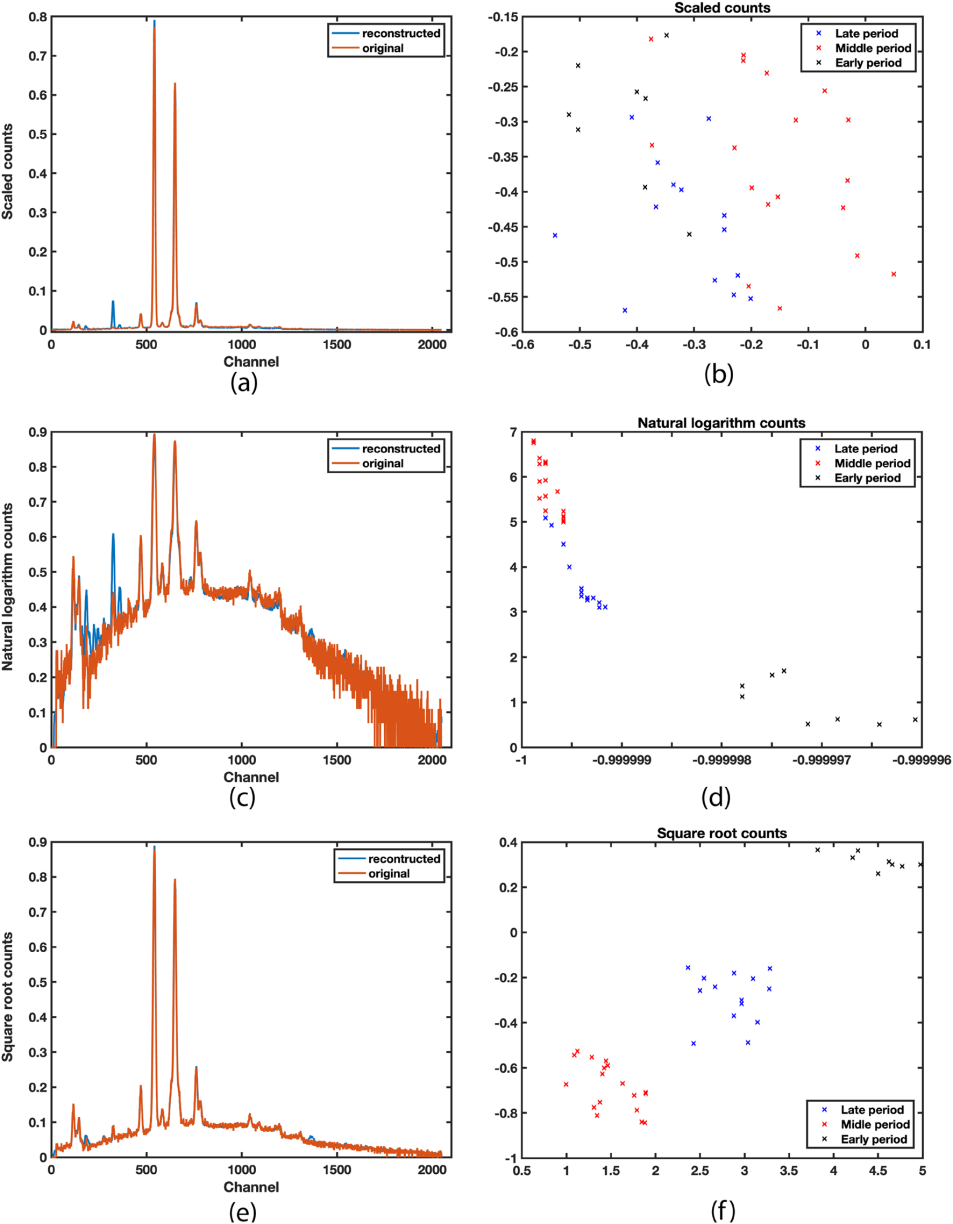
The SAENN training results. (**a**) Raw input spectrum reconstruction. (**b**) Dataset structure in the space of reduced dimensions-the SAENN bottleneck output. (**c**) Natural logarithm-transformed input spectrum reconstruction. (**d**) The SAENN bottleneck output for log-transformed input data. (**e**) Square root transformed input spectrum reconstruction. (**f**) The SAENN bottleneck output for square root-transformed input data.

The training of the above SAENN structure using non-transformed spectral data resulted in highly successful spectrum reconstruction, indicating that essential information about the spectrum was preserved within the bottleneck layer (Fig. 3a). The result of the dimension reduction (Fig. 3b) revealed that dataset structure is rather coherent with small classification possibilities. Since the dimension reduction was performed in an unsupervised manner, and with a closer look at a picture of the reduced space, it can be noticed that some separability could be improved, especially between groups denoted as middle period and rest, as well as groups denoted as late and early period.

As expected, the picture of the dataset in Fig. 3b indicates a small variance in its structure. Knowing that the author used a variety of chemically different yellow pigments, one can expect a higher variance in the data. On the other hand, looking at EDXRF spectra (Fig. 1), small variance can result from the high-intensity lead peaks, which are more times higher than any other. To examine if the information regarding the time attribution lies within the concealed interrelation of elements possessing considerably lower peak intensity, suitable signal spectrum pre-processing methods were performed to highlight the significance of elements with lower peak intensities. The initial approach involved applying natural logarithm operations to the raw spectrum data and training an SAENN. The result of the spectrum reconstruction and its representation in a reduced 2D space can be found in Fig. 3c,d, respectively. As can be seen, the reconstruction was as successful as that conducted on raw data, while the separability was significantly improved. Yet, the result was not satisfactory enough as the two classes (middle and late period) still exhibited small between-class distance, implying a higher possibility of misclassifying unknown data. The result led to the conclusion that perhaps the logarithm function overly emphasized peaks of small intensities, leading to a significant amount of noise that hindered the network’s generalization.

The square root transformation to the raw spectral data was then applied. The idea stemmed from the nature of spectra, which essentially represent the energies of individual elements. The square root transformation aims to derive peak intensity from its energy, effectively condensing the spectrum into a single line. This significantly mitigates the phenomenon where high-intensity elements overshadow others due to applying the sum of squared error algorithm, favoring higher levels. This approach yielded excellent results, as demonstrated in Fig. 3e,f. The reconstruction results of the square root-transformed spectrum are outstanding, showcasing high separability. Data preprocessing substantially assisted in preserving and amplifying information crucial for discerning and distinguishing between different classes.

**Figure 4:**
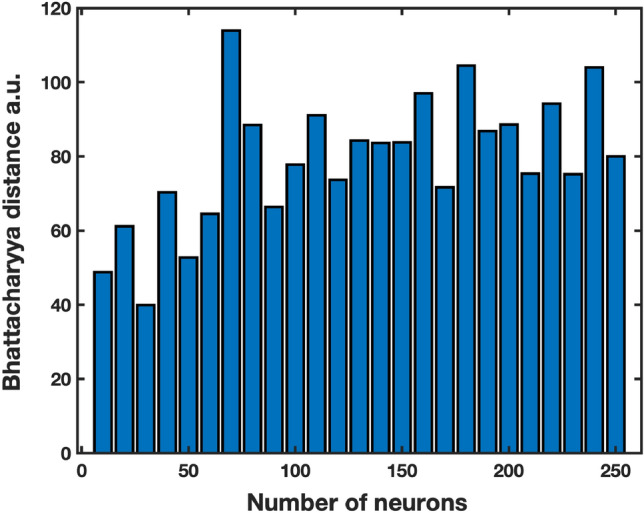
Class separability (measured by the Bhattacharyya distance parameter) depends on the number of neurons in the SAENN structure.

After selecting the network structure, activation functions, and data preprocessing, the optimization of the number of neurons in the intermediate layer of the SAENN was pursued. It was necessary to determine the optimal number of neurons capable of performing a good spectrum reconstruction while ensuring that the bottleneck layer contains coordinates ($x_1$, $x_2$) that provide sufficient separability in the reduced 2D space. If the number of neurons is too small, the network would lack the potential for reconstruction. Conversely, an excessive number of neurons could lead to overfitting, resulting in a non-robust solution with limited applicability. Defining a criterion for evaluating the separability was necessary to determine and optimize the number of neurons. The Bhattacharyya distance was adopted for this purpose. During the training, the number of neurons varied from 10 to 250 in increments of 10. The network underwent 100 training iterations for each selection, and the network providing the highest Bhattacharyya distance was chosen. This extensive repetition was conducted to ensure that the results were independent of the network’s initial random initialization but solely dependent on the desired number of neurons. The result is depicted in Fig. 4, confirming the initial hypothesis about the interdependence between the number of neurons and the mutual separability of classes.

**Figure 5:**
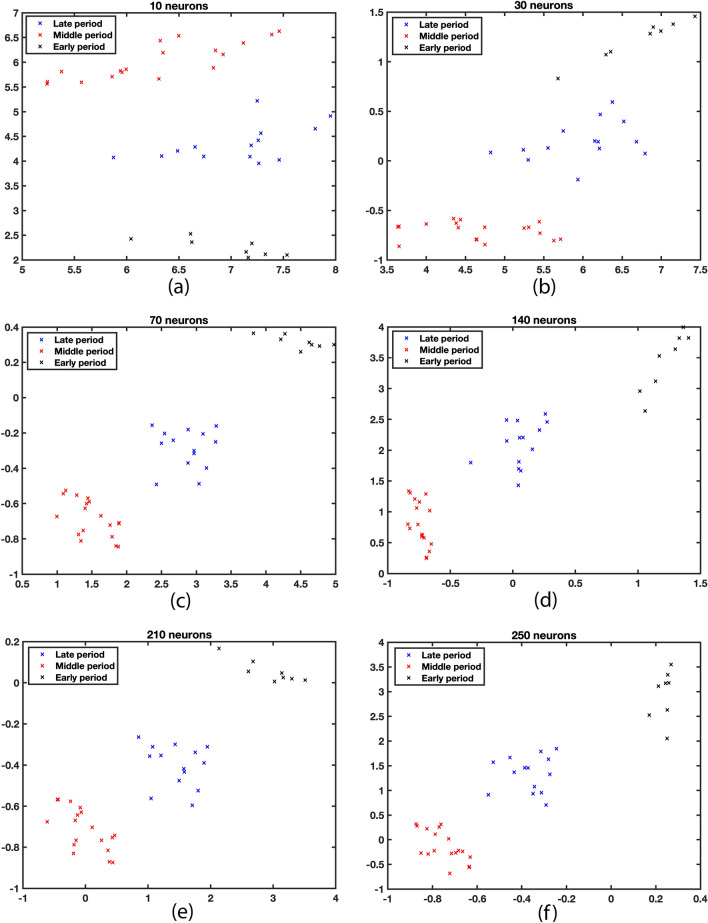
Class separability for the different number of neurons in the encoder/decoder layers.

The class separability depending on the number of neurons is shown in Fig. 5.

As can be seen, the between-class separability increases with the increase of the number of neurons up to 70 in the encoder/decoder layers. This number is a small number of neurons considering the dimension of the input spectrum. At the same time, the increase in group coherence contributes to the higher between-class distance. So, the proposed SAENN enables the management of the two most important factors in the dimension reduction transformations: high class coherence and between-class distance, which allows the best preconditions for successful classification. With a further increase in the number of neurons, the class separability assessment decreases, as seen in Fig. 4. These results can also be observed in Fig. 5d–f, where no significant improvement in class separability can be noticed; in fact, the opposite occurs, regardless of the increased complexity of the SAENN structure. This phenomenon is easily explained by the fact that the network structure becomes too large, leading to the possibility of overfitting. In such cases, it is necessary to have more aggressive measures to prevent overfitting, i.e., training needs to be terminated earlier, which generally results in poorer performance of the trained network. In this way, the optimal number of neurons was identified at 70.

It is important to note that the training and simulations were done on the CPU instead of the GPU. This decision was made after initial experiments, as there was no benefit to running on the GPU due to the small amount of data. Specifically, one measurement is represented by an array of 2048 integer data, and the dataset contains less than 50 recorded spectra. This information is crucial: a high-performance platform is not necessary to apply the proposed procedure, implying a simple implementation and sustainability of the methodology.

## Conclusion

A non-destructive EDXRF analysis of the painted layered materials was performed. The elemental composition of the yellow-colored measured spots was used to attribute artistic paintings to early, middle, and late periods of creation. At the time of the creation of these works of art - the first half of the nineteenth century and beyond, the use of several synthetic yellow pigments of different chemical compositions began. It was established that the author used several other pigments during one period but that she used some of them in the entire oeuvre. These preliminary results suggested using unsupervised techniques to reduce the dimensionality of the initial dataset and examine the possibility of classification by creation time.

Further considerations went in the direction of developing a procedure that would be reliable, and with a short time required for implementation. As the recent application of neural networks has given promising results, especially on synthetic spectra, a proposed method suggests a dimension reduction procedure with the simultaneous maximum reconstruction of the input spectrum based on the SAENN. The neural network was designed so that the hyperparameters were selected in the training process and achieved the maximal reconstruction of the input spectrum. The central layer of this network (bottleneck) has a dimension of 2, so the reduction of dimensions is immediately carried out in a space that provides maximal visualization. The classification success in the reduced space was measured by the between-class distance and within-class scattering using the Bhattacharyya distance parameter. It was found that the SAENN with 70 neurons in encoder/decoder layers and a bottleneck layer with two neurons gives the highest Bhattacharyya distance value. This result is important since satisfactory separability has been achieved with small numbers of neurons, enabling the training to be carried out on the CPU quickly and reliably without expert involvement.

The authors have presented an efficient automated procedure for feature extraction, dimension reduction, and class separation based on the elemental composition of the pigments used and different painting creation periods. In the spirit of modern machine learning technologies, this procedure does not require expert knowledge and can be applied in other areas where the classification of samples is needed based on raw measurements. This reduces expert involvement, and training and processing can be carried out on a conventional CPU in almost real-time. All of this contributes to the automation of time attribution of the work of art, widespread applicability, and sustainability.

## Materials and methods

### Materials

The Serbian artist Katarina Ivanovic was the first educated female painter in modern art history, whose artistic creation began in the first half of the nineteenth century and lasted for more than 40 years. She lived and painted in Hungary, Vienna, Munich, Paris, Zagreb, Belgrade, Nederland, and Italy, meaning that many different materials were available for her work in a long period of painting. The eight oil canvas paintings were selected from the oeuvre to cover three different periods of creation, denoted as early, middle, and late. The details about the analyzed paintings are presented in Table 2. The yellow-colored areas were chosen since the pigments detected during the analysis showed that similar materials were used in all periods of creation, and a variety of newly synthetic materials were available in the period when the author painted.

**Table 2:** The list of the Katarina Ivanovic paintings used for the analysis, the period of creation, and the number of yellow-colored spots per painting.

	Early period		Middle period			Late period		
Painting mark	KI-13	KI-14	KI-8	KI-6	KI-9	KI-3	KI-1	KI-5
Period of creation	1833-1834.	1837.	1847-1850.	1847-1850.	1850.	1865-1870.	1865-1873.	1870-1873.
Number of measurements	3	5	10	1	7	6	4	4

### Portable energy dispersive X-ray fluorescence spectrometry

A total of 40 yellow-colored spots of different nuances were analyzed using portable energy dispersive X-ray fluorescence (pEDXRF) spectrometry. The method was used since the analysis is non-destructive to the painting. The well-established analytical procedure was applied using an in-house developed pEDXRF spectrometer (more details about the instrument can be found in^10,23^).

The experimental setup was as follows: the X-ray tube voltage of 40 kV, the current of $300\, \upmu {\textrm{A}}$, and the measurement time of 120 s. The measurement geometry was as follows: collimated non-filtered incident X-ray beam spot size on the object’s surface of 2 mm, the angle between the incident X-ray beam and the detector of $45^\circ$, the distance from the endpoint of the collimator and detector window and the analyzed surface was 21 mm and 16 mm, respectively. The mentioned parameters were kept constant during all measurements.

The ADMCA software (AMPTEK Inc.) was used for spectra acquisition. The non-original layers on the painted surface were identified using ultraviolet fluorescence photography, enabling analysis of original materials onto sufficiently large spots.

### Autoencoder neural network structure and training

**Figure 6:**
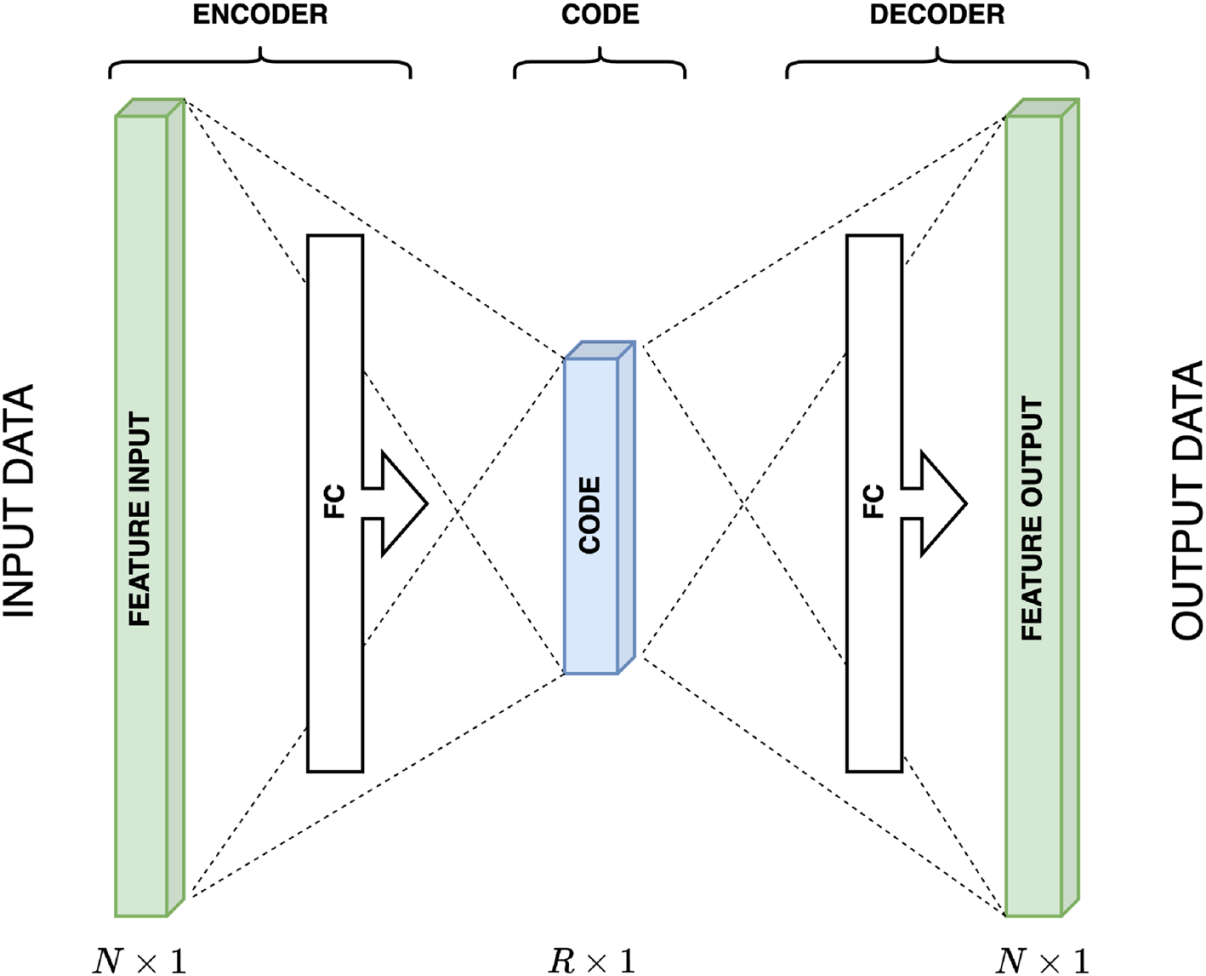
The vanilla autoencoder neural network structure.

A vanilla autoencoder (Fig. 6) is a type of artificial neural network used for unsupervised learning, particularly.

The term vanilla in the context of an autoencoder implies a basic or standard architecture without additional complexities or modifications, focusing on the fundamental structure of an autoencoder. It consists of three primary components: an input layer, a hidden layer (the bottleneck), and an output layer.

- Input Layer. This layer receives the initial input data, which could be images, text, numerical values, or any other type of data that the network is designed to process.
- Hidden Layer (bottleneck). This layer is responsible for learning the compressed or encoded representation of the input data. It has fewer neurons/nodes compared to the input and output layers, forcing the network to find a compressed input representation.
- Output Layer. This layer attempts to reconstruct the input data from the encoded representation generated by the hidden layer. The aim is to create an output closely resembling the input data, effectively learning to reconstruct the original input.

The training process of a vanilla autoencoder involves minimizing the difference between the input and the output (reconstruction) using optimization techniques, often a loss function such as mean squared error (MSE). The network adjusts its weights and biases during the training process to minimize the difference between the input and the output, thereby improving the quality of the reconstructed data. Besides the spectra reconstruction, autoencoder neural network training results in the projection of dataset structure in the low-dimensional space, i.e., unsupervised dimension reduction. The best result of the training is the possibility of attribution, which can be achieved if the grouping in low-dimensional space is considerable. Using autoencoder networks to solve dimension reduction problems requires careful design and selection of many hyperparameters to achieve good, consistent, and sustainable results. These critical hyperparameters encompass the number of layers and neurons in the encoder/decoder layers, the selection of activation functions, the training rate and adaptive mechanisms, the overfitting protection, and the computational platform (CPU or GPU). For the mentioned problem, the hyperparameters were successfully determined by conducting extensive simulations.

### Input data pretreatment

All collected EDXRF spectra were pretreated by the peaks balancing procedure (using the original MATLAB code based on peaks alignment) to minimize any contributions of the experimental setup. However, besides the raw and finely aligned spectral data, the input data were also pretreated in two ways before training the autoencoder neural network. The data was log-transformed (natural logarithm) and square-rooted to examine which method handles small peaks more efficiently. Each analyzed yellow spot represents one 2048-dimensional random vector forming multidimensional space. So, forty 2048-dimensional vectors were used for autoencoder neural network training.

### Class separability measurements

The Bhattacharyya distance $\mu \,(1/2)$ has been used to measure the between-class separability. This parameter is defined as $\mu \,(1/2)=\frac{1}{8} {( M_2-M_1)}^T {\left[ \frac{\Sigma _1+\Sigma _2}{2}\right] }^{-1} ( M_2-M_1 ) + \frac{1}{2} \,\ln \frac{| \frac{\Sigma _1+\Sigma _2}{2}|}{\sqrt{|\Sigma _1| {|\Sigma _2|}' }}$, where $M_1$ and $M_2$ denote expectation vectors, and $\Sigma _1$ and $\Sigma _2$ denote covariance matrices^24^. The high value of this parameter indicates that the two classes are separable enough to avoid misclassification. The parameter $\mu \,(1/2)$ was calculated for all groups formed in the low-dimensional space. The sum of those parameters for pairs of groups in a single autoencoder neural network training and dimension reduction was used as criteria for evaluation.

## Data Availability

The datasets generated and/or analysed during the current study are not publicly available because they contain information about the original materials used and can be used to copy works of art but are available from the corresponding author on reasonable request.
